# Cortical Surface Thickness in the Middle-Aged Brain with White Matter Hyperintense Lesions

**DOI:** 10.3389/fnagi.2017.00225

**Published:** 2017-07-17

**Authors:** Ying Zhuang, Xianjun Zeng, Bo Wang, Muhua Huang, Honghan Gong, Fuqing Zhou

**Affiliations:** ^1^Department of Oncology, The Second Hospital of Nanchang City Nanchang, China; ^2^Department of Radiology, The First Affiliated Hospital, Nanchang University Nanchang, China

**Keywords:** white matter hyperintense lesion, cortical surface thickness, normal-appearing gray matter, voxel-based morphometry, aging

## Abstract

**Background and purpose**: Previous voxel-based morphometry (VBM) studies have suggested that cortical atrophy is regionally distributed in middle-aged subjects with white matter hyperintense (WMH) lesions. However, few studies have assessed cortical thickness in middle-aged WMH subjects. In this study, we examined cortical thickness as well as cortical morphometry associated with the presence of WMH lesion load in middle-aged subjects.

**Participants and methods**: Thirty-six middle-aged subjects with WMH lesions (WMH group) and without clinical cognitive impairment, and 34 demographically matched healthy control subjects (HCS group) participated in the study. Cortical thickness was estimated using an automated Computational Anatomy Toolbox (CAT12) as the distance between the gray-white matter border and the pial surface. Individual WMH lesions were manually segmented, and WMH loads were measured. Statistical cortical maps were created to estimate differences in cortical thickness between groups based on this cortex-wide analysis. The relationship between WMH lesion loads and cerebral cortical thickness was also analyzed in CAT12.

**Results**: Cortical thickness was significantly lower in the WMH group than in the controls in multimodal integration regions, including the right and left dorsal anterior cingulate cortex (dACC), right and left frontal operculum (fO), right and left operculum parietale (OP), right and left middle temporal gyrus (MTG), and left superior temporal gyrus (STG; *P* < 0.01, family-wise error (FWE)-corrected). Additionally, cortical thickness was also lower in the recognition regions that contained the right temporal pole (TP), the right and left fusiform gyrus, and the left rolandic operculum (RO; *P* < 0.01, FWE-corrected). The results revealed that in the left superior parietal lobule (SPL), cortical thickness was higher in the WMH group than in the HCS group (*P* < 0.01, FWE-corrected). A voxel-wise negative correlation was found between cortical thickness and WMH lesion loads in the right orbitofrontal cortex (OFC), right dorsolateral prefrontal cortex (DLPFC), and right subcallosal cortex (*P* < 0.01, FWE-corrected).

**Conclusion**: The main findings of this study suggest that middle-aged WMH subjects are more likely to exhibit cortical thinning, especially in multimodal integration and recognition- and motor-related regions. The current morphometry data provide further evidence for WMH-associated structural plasticity.

## Introduction

Assessments of subtle structural differences or changes in the gray matter that use non-invasive imaging methods have provided insights into the mechanisms underlying both normal brain aging and pathological processes, including middle-aged patients with white matter hyperintense (WMH) brain lesions. In individuals, studies of the longitudinal progression of WMH lesions have suggested that they have a vascular origin and are associated with vascular risk factors, as shown on brain fluid-attenuated inversion recovery (FLAIR) or T2-weighted imaging (T_2_WI). These lesions are associated with neuronal loss, demyelination, and gliosis on neuropathological examination, and they have been linked to cerebral hypo-perfusion and reduced WM integrity (Debette and Markus, 2010; Grueter and Schulz, 2012; Maniega et al., 2015). It is generally accepted that WMH typically increase with both normal aging and the progression of cerebral microvascular disease. However, WMH lesions are not common in healthy middle-aged brains (Valdés Hernández Mdel et al., 2013). Hence, the recognition of sporadic WMH lesions in middle-aged brains is often neglected by radiology or neurology personnel because they lack specific clinical symptoms’ correlation in most subjects (Soriano-Raya et al., 2012). Previous voxel-based morphometry (VBM) studies have suggested that cortical atrophy is regionally distributed in middle-aged subjects with WMH lesions (Peng et al., 2016). However, few studies have investigated cortical thickness in middle-aged WMH subjects. In cortical thickness mapping, which is different than VBM, thickness is calculated as the distance between the white matter–gray matter (WM–GM) surface (approximately 1.6–4.5 mm; Dahnke et al., 2013). This measurement provides specific information about neuronal loss or degradation, which is indicated by thinning of the cortex (Yotter et al., 2011b; Dahnke et al., 2013). Cortical thickness mapping has been shown to be more sensitive to age-related decline than VBM, which uses a fully automated method based on the projection-based thickness (PBT) measurement (Dahnke et al., 2013) to provide a local measure of GM within the cortex (Lemaitre et al., 2012). No study has directly assessed the effect of WMH lesions on the cortical thickness measurements provided by a surface-based reconstruction approach.

Given the previous finding in aging (Debette and Markus, 2010; Arvanitakis et al., 2016) and middle-aged subjects (Peng et al., 2016), we hypothesized that sporadic WMH lesion also contributes to the etiology of disrupted microstructural integrity resulting to a reduction of cortical thickness in normal-appearing GM in the middle-aged subjects. Therefore, in the present study, we examined WMH-related changes in cortical thickness across all cortical regions in the brain of middle-aged individuals with WMH lesions (WMH group). Additionally, we also analyzed the relevance of the results obtained here using automated cortical thickness analysis to the data currently available in the literature in healthy aging patients and those with neurodegenerative processes.

## Materials and Methods

This case-control study was performed according to the approved guidelines of the Medical Research Ethics Committee and the Institutional Review Board of the First Affiliated Hospital of Nanchang University and conducted in compliance with the principles of the Declaration of Helsinki. Written informed consent was obtained from all subjects in the study.

### Subjects

**Figure 1:**
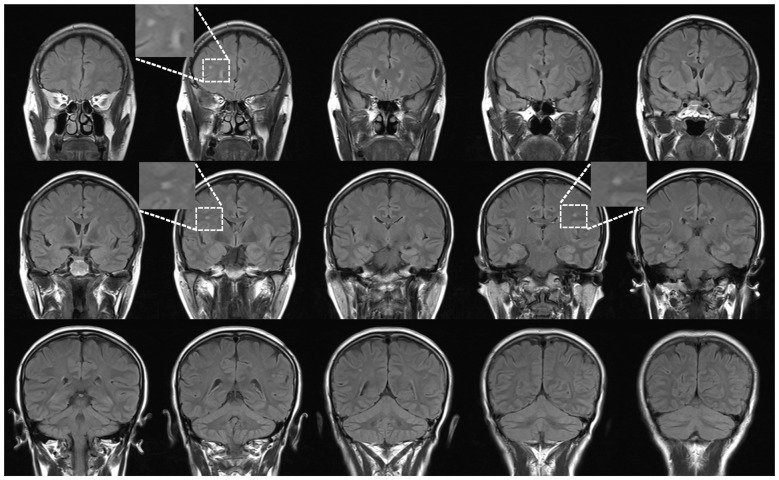
An example case: coronal view of fluid-attenuated inversion recovery (FLAIR) in a 46-year-old female subject. All participants with sporadic white matter hyperintense lesions underwent a health examination.

Thirty-eight subjects were sequentially recruited based on the presence of a WMH finding (an example is shown in Figure 1) on T_2_WI and FLAIR imaging from August 2012 to July 2014 at The First Affiliated Hospital of Nanchang University. We previously described this sample population in an analysis using VBM (Peng et al., 2016). The following inclusion criteria were applied: (1) aged 45–59 years old; (2) a physical health examination performed in a subject who was not a medical patient with sporadic WMH lesions; (3) a preference for individuals from the urban community of Nanchang city who agreed to follow-up including cognitive and other medical tests; and (4) no history of neurologic symptoms or signs and laboratory screening. The following exclusion criteria were applied: (1) probable Alzheimer’s disease diagnosis; (2) history of overt stroke (cortical infarcts), hypertension (systolic blood pressure >140 mmHg or diastolic blood pressure >90 mmHg), diabetes, and the notch-3 mutation in cases with suspected subcortical infarcts and leukoencephalopathy (CADASIL) as determined by testing; (3) small vessel-related systemic disease or organ failure, including renal, heart and respiratory failure; (4) cognitive irregularities according to a Mini-Mental State Examination (MMSE) screening (a score lower than 27); (5) not having a known contraindication to MRI scanning (i.e., severe claustrophobia, cardiac pacemaker, or metallic implant likely to contribute significant artifact to images); or (6) unqualified image data.

The healthy control subjects (HCS) were demographically matched by sex, age and education level from respondents to flyers who were randomly recruited from the urban community of Nanchang city. All recruited individuals lacked a history of neurological or psychiatric disorders and any evidence of WMH lesions in T_2_WI and FLAIR imaging. The MMSE was also used as a cognitive screening tool in these subjects. Finally, body mass index (BMI), which was defined as body mass divided by the square of body height, was determined for all recruited individuals as a simple record for obesity.

### MRI Data Acquisition

All MRI data were acquired using a 3-TeslaTrio MR imaging scanner system (Trio Tim, Siemens Medical Systems, Erlangen, Germany). These included conventional T_2_WI and FLAIR imaging for diagnoses and radiological evaluations as well as high-resolution T_1_WI for cortical surface complexity analyses. The following imaging parameters were used in the study: (1) turbo spin echo-imaging sequence for T_2_WI scans: repetition time/echo time = 5100/117 ms, field of view = 240 × 240 mm, matrix = 416 × 416, number of excitations = 3, echo train length = 11, 22 axial slices with a 6.5-mm thickness; (2) FLAIR imaging: repetition time/echo time/inversion time = 7000/79/2500 ms, 50 slices, 240 × 217 matrix, 0.43 × 0.43 × 2 mm^3^ voxels, 3:42 min; and (3) three-dimensional high-resolution T_1_WI: repetition time/echo time = 1900/2.26 ms, field of view = 215 × 230 mm, matrix = 240 × 256, number of excitations = 1, and 176 sagittal slices with a 1.0-mm thickness.

### Preprocessing and Cortical Surface Extraction

The MRI data were visually inspected for obvious artifacts arising from subject motion and instrument malfunction. The high-resolution T_1_WI was preprocessed and the cortical surface was extracted using automated procedures in Computational Anatomy Toolbox (CAT12)^1^ within SPM12 while running MATLAB 8.4 (R2014b; MathWorks, Natick, MA, USA). Briefly, T1 images were bias-field corrected, skull-stripped, aligned to a Montreal Neurological Institute standard space (MNI-152 template), and classified as GM, WM, or cerebrospinal fluid, all within the same generative model (Kurth et al., 2015). To improve registration accuracy, the DARTEL algorithm was used to create a group-specific template and calculate the individual non-linear transformation to this template in SPM8.

In CAT12, a new fully automated method allows cortical thickness to be measured and the central surface to be reconstructed in a single step (Dahnke et al., 2013). The program uses tissue segmentation to estimate the WM distance and projects the local maxima (which is equal to the cortical thickness) to other GM voxels using a neighbor relationship that is described by the WM distance. This PBT allows partial volume information, sulcal blurring, and sulcal asymmetries to be managed without explicit sulcus reconstruction via skeleton or thinning methods. For inter-subject comparisons, local complexity maps were re-parameterized into a common coordinate system and smoothed using a 15-mm Gaussian heat kernel (Yotter et al., 2011a; Madan and Kensinger, 2016). For quality control, we excluded two WMH subjects and four healthy controls based on the poor quality of their cortical surface reconstructions.

### Measurement of WMH

#### WMH Scores

WMH lesions were scored in FLAIR images using the age-related white matter changes (ARWMC) scale (range, 0–30; Wahlund et al., 2001; Kapeller et al., 2003). The ARWMC records five different regions (the frontal, parieto-occipital, temporal, infratentorial regions and the basal ganglia) from the right and left hemispheres and uses a 4-point range (0–3). To ensure the reliability of these measurements, one expert neuroradiologist (FZ) performed all ratings after being trained using a standard data set. Another senior neuroradiologist (HG) cross-checked a random sample that consisted of 30% of the ratings.

#### Volumetric Assessment

The procedure used to obtain a volumetric measurement of WMH lesions has been previously described (Charil et al., 2007; Shu et al., 2011). The main process is briefly described here. Individual WMH lesions were manually delineated on the FLAIR images (FZ), and the lesion fraction was defined as the ratio between the lesion volume of the individual space and the ICV. We then obtained a binary lesion mask that was normalized to the MNI space to remove head size from the lesion volume calculation, which was re-measured on two separate occasions (at least 3 months apart) as a quality control. Inter-rater reliability was 92.3%.

Brain parenchymal fraction (BPF) is a simple and easy to calculate measurement in CAT12 and was used as an index of the extent of global cerebral atrophy. The BPF was calculated as the brain parenchyma divided by the total brain intracranial volume (formula 1) and was used to control for variability in head size.

(1) BPF=Volume of (GM+WM)/Volume of (GM+WM+Cerebrospinal Fluid)

#### Statistical Analysis

First, group differences in global cortical thickness were compared between the WMH and HCS groups using a two-sample *t*-test. Then, regional differences were compared using a general linear model in the statistical analysis menu of CAT12, and these results were used as an indication of WMH-related alterations. A threshold of *P* < 0.01 and a family-wise error (FWE) correction were applied.

A two-sample *t*-test was performed to compare group differences in age, education, BMI, MMSE, BPF and global mean thickness in Statistical Product and Service Solutions V13.0 (SPSS Inc., Chicago, IL, USA). In addition, a Chi-square test was used for comparisons of gender and handedness.

Finally, we assessed the relationship between WMH lesion load and cerebral cortical thickness in the WMH group using a simple linear regression model for the correlation analysis in CAT12. Age, gender and education level were used as covariates of no interest. The threshold was set at a significance level of *P* < 0.01 and was FWE-corrected for multiple comparisons.

## Results

### Demographic and Clinical Description

Thirty-six middle-aged subjects with WMH lesions (WMH group) and 34 well-matched HCS were chosen for the study. The two groups were similar in age, gender, education level and handedness (Table 1). Neuropsychological test (MMSE) scores confirmed that the groups had normal cognitive functions did not differ significantly between the WMH and HCS groups (29.25 ± 1.08 vs. 29.65 ± 0.73). The mean total ARWMC score in record five regions was 6.389 ± 4.291 (in numbers) indicating a slight load of WMHs, and a low lesion volume was recorded (3.616 ± 1.436 ml) in the WMH group. Together, these indicate mild incomplete ischemia related to cerebral small vessel arteriolosclerosis. The structural assessment for the WMH group is also presented in Table 1. No significant difference was detected in the BPF and global mean thickness between the two groups.

**Table 1:** Demographic and clinical characteristics of the WMH and HCS groups.

Characteristic	WMH group (*n* = 36)	HCS (*n* = 34)	*t* (P) values
Age, years	54.75 ± 8.15	51.58 ± 7.13	1.730 (0.088)
Gender (male/female)	16/20	16/18	0.048 (0.826)^#^
Education, years	12.08 ± 3.51	12.21 ± 3.22	−0.152 (0.879)
Handedness, right/left	36/0	34/0	–^#^
BMI (kg/m^2^)	22.69 ± 1.31	22.85 ± 1.23	−0.521 (0.604)
MMSE	29.25 ± 1.08	29.65 ± 0.73	−1.790 (0.078)
BPF	0.772 ± 0.024	0.774 ± 0.021	−0.390 (0.682)
WMH scores (ARWMC)	6.389 ± 4.291	–	–
Lesion volume (ml)*	3.616 ± 1.436	–	–
Lesion fraction	(2.62 ± 1.06) × 10^−3^	–	–
Global mean thickness	2.609 ± 0.102	2.616 ± 0.089	−0.307 (0.760)

Note: values shown are mean ± SD unless noted otherwise. Lesion volume was calculated in a “Montreal Neurological Institute” space. WMH, white matter hyperintensities; HCS, healthy control subjects; BMI, body mass index; MMSE, Mini-Mental State Examination; BPF, brain parenchymal fraction; ARWMC, age-related white matter changes; * = measurement of individual space; ^#^ = Chi-square test; – = no report. The same abbreviations are used for all figures and tables.

### Group Comparison of Regional Cortical Thickness

**Figure 2:**
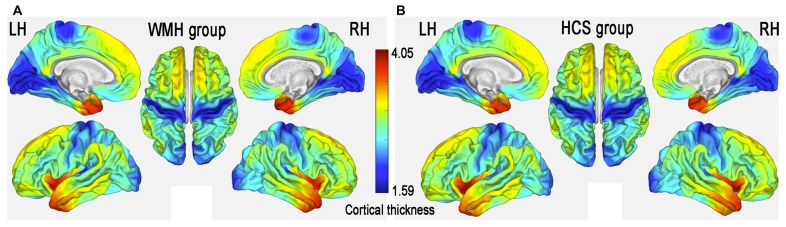
Mean cerebral cortical thickness patterns in middle-aged subjects with white matter hyperintense (WMH) lesions (WMH group) **(A)** and healthy controls (HCS) **(B)**.

The patterns of mean cerebral cortical thickness are shown in Figure 2 for the WMH group (Figure 2A) and the HCS group (Figure 2B). Both groups exhibited a thicker cortex in the temporal pole (TP), insula and dorsolateral prefrontal cortex (DLPFC), while thinning was evident in the visual cortex and around the central sulcus regions. On visual inspection, although the two groups showed similar patterns of cortical thickness, the WMH group seemed to have lower cortical thickness in the anterior cingulate cortex (ACC) and operculum parietal (OP) regions.

**Figure 3:**
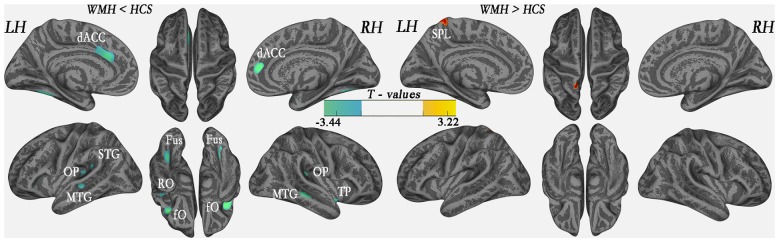
Comparison of local cortical thickness between the WMH group and the HCS group (*P* < 0.01, family-wise error (FWE)-corrected). Indigo indicates a significantly lower cortical thickness value, and yellow indicates a significantly higher cortical thickness value.

The global mean cortical thickness was not significantly different (*t*_(68)_ = −0.307; *P* = 0.760) between the WMH (2.609 ± 0.102) and control groups (2.616 ± 0.089). However, in the multimodal integration regions that contained the right and left dorsal ACC (dACC), right and left frontal operculum (fO), right and left OP, right and left middle temporal gyrus (MTG) and the left superior temporal gyrus (STG), regional cortical thickness was lower in the WMH group than in the HCS group. Additionally, cortical thickness was lower in the recognition regions that contained the right TP, including the left and right fusiform gyrus and the left rolandic operculum (RO; *P* < 0.01, FWE-corrected; Figure 3 and Table 2). These results reveal that regional cortical thickness is higher in the left superior parietal lobule (SPL) in the WMH group than in the HCS group (*P* < 0.01, FWE-corrected; Figure 3 and Table 2).

**Table 2:** Comparison of regional cortical thickness between the WMH group and HCS.

Anatomical location		Brodmann area	MNI space (X, Y, Z in mm)	Cluster size (voxels)	Peak-*t* values	Peak-*P* values
**WMH < HCS**						
dACC	RH	32	(13, 46, 12)	354	−3.44	0.001
dACC	LH	24	(−9, 25, 27)	934	−3.33	0.001
fO	RH	40	(39, 17, 9)	750	−3.37	0.001
fO	LH	48	(−38, 23, 8)	677	−3.90	0.000
TP	RH	36	(40, 2, −21)	385	−2.84	0.003
OP	LH	48	(−40, −29, 12)	248	−2.87	0.003
RO	LH	48	(−45, −8, 9)	252	−2.83	0.003
OP	RH	41	(40, −34, 14)	224	−2.71	0.004
STG	LH	41	(−46, −40, 18)	202	−2.87	0.003
MTG	RH	21	(63, −30, −8)	410	−3.07	0.002
MTG	LH	21	(−51, −26, −5)	290	−3.04	0.002
Fusiform gyrus	LH	37	(−32, −51, −18)	492	−3.35	0.001
Fusiform gyrus	RH	37	(28, −57, −16)	193	−2.74	0.004
**WMH > HCS**						
SPL	LH	40	(−13, −53, 70)	524	3.22	0.001

*Abbreviations: dACC, dorsal anterior cingulate cortex; fO, frontal operculum; HCS, healthy control subjects; LH, left hemisphere; TP, temporal pole; ITG, inferior temporal gyrus; MNI, Montreal Neurological Institute; MTG, middle temporal gyrus; OP, parietal operculum; RH, right hemisphere; RO, rolandic operculum; STG, superior temporal gyrus; SPL, superior parietal lobule; WMH, white matter hyperintensity. The same abbreviations are used for all figures and tables*.

### Correlational Analyses between Regional Cortical Thickness and Lesion Loads

**Figure 4:**
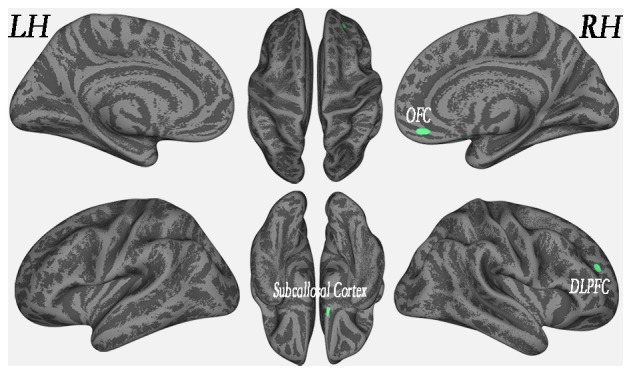
Regional cortical thickness was correlated with lesion load in the WMH group (*P* < 0.01, FWE-corrected).

In the WMH group, cortical thickness and “lesion fraction” scores were negatively correlated with the right orbitofrontal cortex (OFC), right DLPFC and right subcallosal cortex (*P* < 0.01, FWE-corrected; Figure 4 and Table 3). We have also presented a flexible statistical result, as shown in Supplementary Figure S1.

**Table 3:** Association between regional cortical thickness and lesion load in the WMH group (*P* < 0.01, FWE-corrected).

Anatomical location		Brodmann area	MNI space (X, Y, Z in mm)	Cluster size (voxels)	Peak-*t* values	Peak-*P* values
OFC	RH	11	(8, 42, −13)	107	−3.01	0.002
DLPFC	RH	46	(24, 45, 22)	98	−2.86	0.004
Subcallosal cortex	RH		(9, 10, −18)	90	−2.86	0.004

*Abbreviations: OFC, orbitofrontal cortex; DLPFC, dorsolateral prefrontal cortex*.

## Discussion

In the present study, we investigated cortical thickness and its relationship with WMH lesion load in middle-aged WMH subjects. We found that patients in the WMH group were more likely to exhibit cortical thinning in certain areas, including multimodal integration, recognition and language-processing regions, than individuals in the HCS group. These results revealed that cortical thickness was higher in the left SPL in the WMH group than in the HCS group. Additionally, we found that WMH lesion load and cortical thickness were significantly correlated in the right OFC, right DLPFC and right subcallosal cortex.

### Regional Decrement in Cortical Thickness in the WMH Group

Cerebral WMH lesions are important structural abnormalities in the aging brain (Lemaitre et al., 2012; Valdés Hernández Mdel et al., 2013; Maniega et al., 2015). However, they are generally ignored in “normal” middle-aged subjects or dismissed as unimportant imaging findings. In a meta-analysis of longitudinal studies (Debette and Markus, 2010), WMH lesions in aging brains were clearly associated with progressive cognitive impairments (Debette and Markus, 2010), physical dysfunction (e.g., abnormal gait (de Laat et al., 2011) and disturbed balance (Baezner et al., 2008)), and a risk of late-onset depression (Arvanitakis et al., 2016). Recently, a relationship was noted among WMH lesion load and regional cortical atrophy and dysfunction in a community sample of middle-aged participants (Soriano-Raya et al., 2012; Peng et al., 2016). However, the association between WMH lesions and cortical thickness remains unclear.

In the present study, significant thinning was also observed in the multimodal integration regions of the cortex, including the right and left dACC, right and left fO, right and left OP, right STG and right and left posterior MTG, in middle-aged WMH subjects. In studies of aging, these multimodal integration regions frequently exhibited GM loss (Lemaitre et al., 2012) and were susceptible to WMH-related GM loss (Maniega et al., 2015; Peng et al., 2016). Multimodal integration, also known as multisensory integration, is associated with different sensory modalities, and the interactions between the multimodal integration centers of the brain are central to adaptive behavior. For example, the dACC is a brain region that subserves motor control to guide voluntary behavior (Asemi et al., 2014) and cognition and involves reward-based decision making (Bush et al., 2002) and mediates the expression of fear (Milad et al., 2007) and ongoing behavioral adaptations (Sheth et al., 2012). The operculum includes the fO (Higo et al., 2011) and the OP (Eickhoff et al., 2006). The fO plays a role in controlling cognitive processes (Higo et al., 2011), and the OP is responsible for integrated sensorimotor processing and action control (Eickhoff et al., 2006). Recent studies have revealed the multisensory processing capabilities of the STG (Senkowski et al., 2008), which are merged with the visual-auditory processing capability of the posterior MTG (a direct connection; Sepulcre et al., 2012). The AI is thought to be responsible for emotions (Cacioppo et al., 2013). Several thinned temporal regions were observed in this study. These thinned regions are involved in processing sensory input to produce derived meanings related to the appropriate retention of visual memories, language comprehension, and emotional association (Hickok and Poeppel, 2007; Pertzov et al., 2013). In this study, thinned multimodal integration regions were slightly correlated with lesion loads in the left OP. These data collectively indicate that the atrophy of the OP is a potentially important contributor to the declining interactions between sensory, motor and cognitive regions in middle-aged WMH subjects. Although it needs to be confirmed in future studies.

In the WMH group, we observed a significant decrease in the thickness in the language-processing region (left RO). Declines in language-processing functions are common in the elderly (Carp et al., 2011) and have been associated with the loss of integrity in brains with WM lesions and those with normal-appearing WM (Baezner et al., 2008; de Laat et al., 2011). However, it was not possible to rule out an association between language-skills and the thickness of the RO in our study. The MMSE, which has four items that score language, was applied to the subjects, but there was no significant correlation with the cortical thickness of the left operculum (*P* = 0.886). WMH lesions should be addressed as a damaging factor in the RO cortex in a future study of “clinically normal” middle-aged subjects.

We also detected significant thinning in the recognition region of the left and right fusiform gyri. The fusiform gyrus has been linked with various neural pathways related to recognition, including synesthesia, dyslexia and prosopagnosia (Weiner and Zilles, 2016). Reduced cortical volume and an increase in subcortical WMH lesions in the fusiform gyrus have been reported to be associated with age-related augmentation in tissue-preserving functions (Gunning-Dixon and Raz, 2003) and compensated regional cerebral blood flow (rCBF), leading to reduced efficacy of interregional neural communications as a result of WM deterioration (Kraut et al., 2008). The fusiform gyrus should therefore be investigated in future studies of middle-aged WMH subjects.

### Regional Increases in Cortical Thickness in the WMH Group

Our results suggest that the left SPL has a higher cortical thickness in the WMH group. The larger cortical thickness of the SPL could result from the swelling of neurons, which can cause ischemic hypoxia or inflammatory demyelination, or differences between groups that can be accounted for by a number of factors, including normal variation, age and other risk factors. In a VBM study, an increase in GM density was observed near thinning regions, indicating the presence of a compensatory mechanism in brain regions exhibiting mild damage (Peng et al., 2016). Moreover, a regional increase in cortical thickness might reflect a gain of function or compensatory “hypertrophied” area in the context of an acquired brain lesion (i.e., WMH). For example, changes in plasticity in individuals with an acquired brain injury (whether it be ischemic, neoplastic, primary neurodegenerative or traumatic) most frequently lead to modifications in functional brain networks, which have been associated with distinct patterns of sensory-motor, behavioral or cognitive impairments; with its sparing (García et al., 2017) or even with the gaining of new skills (such as arts; Seeley et al., 2008). The SPL is involved in spatial orientation, visual and hand sensory information input, and other general functions of the parietal lobe. In this study, the middle-aged WMH subjects did not report subjective or objective signs of functional damage. However, the finding of increased cortical thickness in the left SPL is interesting. If a compensatory mechanism could be confirmed in this region, it could be used to help prevent WMH-related degeneration. Moreover, cortical structural reconstruction has rarely been reported in aging individuals with WMH lesions. Hence, the results of this study provide an opportunity to improve our understanding of the pathogenic mechanisms involved in age-related small vessel disease.

### Regional Cortical Thickness-Associated WMH Lesion Load

In the WMH group, cortical thickness and “lesion fraction” scores in the right OFC, right DLPFC and right subcallosal cortex were slightly negatively correlated. Previous VBM studies have suggested that the cortical atrophy that is associated with WMH lesion load is regionally distributed in middle-aged subjects (Peng et al., 2016). Earlier pathology reports that focused on demyelination and axonal loss in WMH subjects described these changes as “ischemic”. Extensive WMH lesions have also been associated with a loss of cortical thickness of glia, vacuolation, a loosening of WM fibers, and myelin loss. The negative correlation between WMH load and prefrontal (orbito- and dorsolateral-) and subcallosal (subgenual, which is also part of the ACC) cortical thickness can be viewed as an additional way to alter high-order cognitive nodes, which themselves serve to integrate information processed not only from multimodal sensory-motor and perceptual brain networks but also from behavioral, affective, social cognitive and internal body sensation inputs (this latter process is also known as interoception).

Regarding the limitations of this study, the middle-aged WMH subjects who were included were sporadic, recruited during a high-hierarchy health examination at a university hospital, and had normal cognitive status. They do not, therefore, represent a community-dwelling population. All of the middle-aged WMH subjects reported a very good experience, which was confirmed by their high MMSE scores (29.25 ± 1.08). The MMSE is both a valid and reliable method for measuring cognitive impairment. Few tools have been designed and validated to test the general cognitive domains, partly because there is a lack of suitable local versions during the study stage. In future studies, it would useful to apply more suitably evaluated tools to screen for impairments in this WMH population. Other limitations of the study include its very small sample size, our inability to determine temporal causality because of the study design (i.e., case-control design), and a lack of risk factor data to include as covariates in the multivariable regression models.

## Conclusion

In this study, we observed several thinning cortical regions, consistent with the findings reported in a previous study of GM density abnormalities in middle-aged WMH subjects. These results increase our understanding of the structural modifications involved in alterations of cortical thickness and how they can be, to a certain extent, associated with WMH lesion load.

## Author Contributions

FZ designed the study. BW and MH acquired the data. YZ, XZ and FZ processed the neuroimaging data. HG performed the statistical analyses. All authors contributed to data interpretation and writing the article.

## Conflict of Interest Statement

The authors declare that the research was conducted in the absence of any commercial or financial relationships that could be construed as a potential conflict of interest.

## Footnotes

^1^http://dbm.neuro.uni-jena.de/cat/

## Supplementary Material

FIGURE S1 Regional cortical thickness was correlated with the lesion loads in the white matter hyperintense (WMH) group (**A**: *P* < 0.05, without corrected; **B**: *P* < 0.01, without corrected).
