# Supplementary figures and images for: Cortical Surface Thickness in the Middle-Aged Brain with White Matter Hyperintense Lesions
Source: Front Aging Neurosci. 2017 Jul 17;9:225. doi: 10.3389/fnagi.2017.00225 (PMC5511819; doi:10.3389/fnagi.2017.00225)

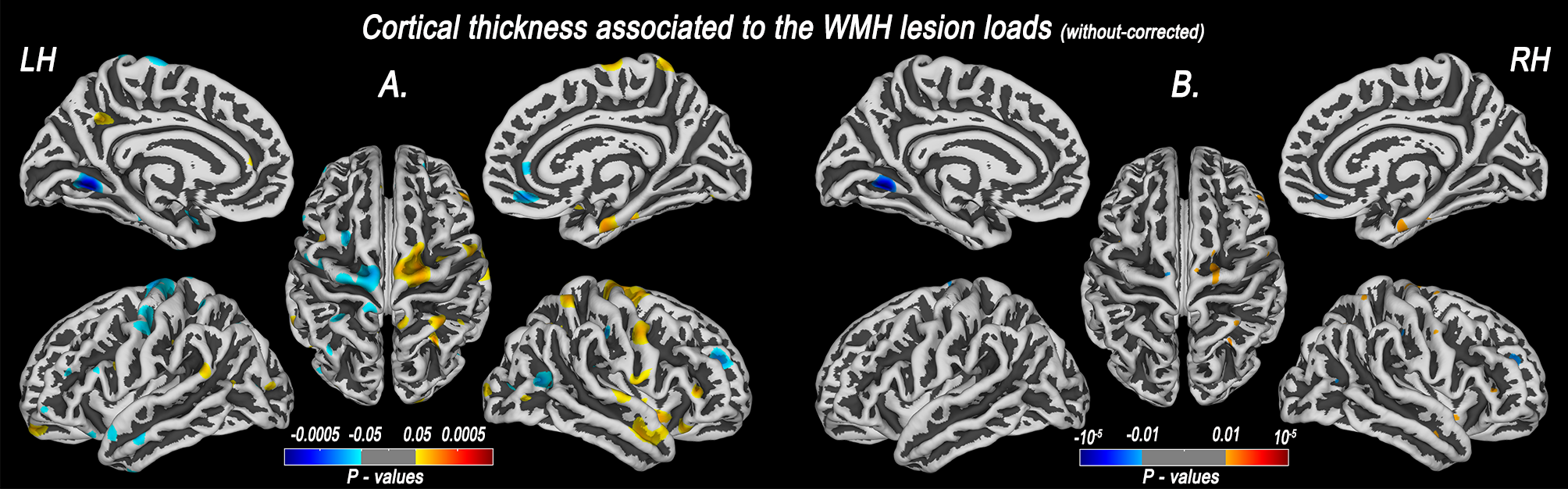

Supplement: FIGURE S1 — Regional cortical thickness was correlated with the lesion loads in the white matter hyperintense (WMH) group (A: P < 0.05, without corrected; B: P < 0.01, without corrected). [file Image_1.tif]
